# *VDR* Gene Polymorphisms in Healthy Individuals with Family History of Premature Coronary Artery Disease

**DOI:** 10.1155/2021/8832478

**Published:** 2021-01-29

**Authors:** Martyna Fronczek, Joanna Katarzyna Strzelczyk, Tadeusz Osadnik, Krzysztof Biernacki, Zofia Ostrowska

**Affiliations:** ^1^Department of Medical and Molecular Biology, Faculty of Medical Sciences in Zabrze, Medical University of Silesia, Katowice 40-055, Poland; ^2^Department of Pharmacology, Faculty of Medical Sciences in Zabrze, Medical University of Silesia, Katowice 40-055, Poland; ^3^Second Department of Cardiology and Angiology, Silesian Centre for Heart Diseases, Zabrze, Poland

## Abstract

**Aim:**

The gene encoding the vitamin D receptor (*VDR*) is considered in many studies to be a good candidate responsible for susceptibility to several diseases such as coronary artery disease (CAD). Epidemiological data show that cardiovascular disease is one of the major health problems in Polish society. Basic studies show that genetic factors play a significant role in the pathogenesis of CAD. We conducted this clinical study to determine if the *VDR* gene polymorphisms TaqI (rs731236), ApaI (rs7975232), and FokI (rs2228570) could predispose healthy individuals to an increased risk of premature CAD (P-CAD) incidents.

**Methods:**

We genotyped 845 subjects in a cohort consisting of 386 healthy volunteers with a documented P-CAD incident in their first-degree relatives and 459 healthy volunteers without family history (FH) of P-CAD. TaqI, ApaI, and FokI polymorphisms in *VDR* were genotyped using TaqMan assays and the endpoint genotyping method (qPCR). Statistical analyses were performed using the Power Analysis Software STATISTICA v.13.3.

**Results:**

Although no statistical significance was found for TaqI and ApaI genotype frequencies, the AA genotype of FokI polymorphism was significantly more frequent in the study group compared to the control group (24.61% vs. 16.99%). The results of logistic regression analysis suggested a significant association between FokI polymorphism and FH of P-CAD in heathy people under the recessive model (OR: 1.26 (1.07-1.49, *p* = 0.007)); however, the frequency of *VDR* haplotypes did not differ significantly between the control and study populations.

**Conclusions:**

FokI polymorphism is may be associated with FH of P-CAD. FokI polymorphism may predispose to the development of P-CAD among healthy people over the next years.

## 1. Introduction

Coronary artery disease (CAD) is one of the most commonly diagnosed diseases in industrialized and developing societies [1]. Lipid disorders, enhanced by bad eating habits along with reduced physical activity and genetic predisposition, may have an impact on development of CAD at a young age [1, 2]. Premature CAD (P-CAD) is defined as an ischemic heart disease that occurs in women before the age of 65 and in men before the age of 55 [3–5]. Heart and vascular diseases are still among the main health problems concerning Polish society; according to statistical data of the National Database of Heart Attacks AMI-PL 2009-2012, they accounted for 28% of deaths in 2009 among people in the age of professional activity (20-64 years). Despite improvement of living situations and increase in awareness over recent years, mortality rates due to these diseases are still too high in Poland, and data compiled in 2014 estimate that the number of deaths from cardiovascular disease in 2020 will exceed 200,000 [6]. Clinical studies show that genetic factors play a significant role in pathogenesis of CAD, and it is estimated that they may cause up to 60% of cases [7–9].

Epidemiological studies show that around one billion people worldwide suffer from vitamin D (VitD) deficiency [10, 11]. This high frequency may be due to limited exposure to sun, use of sunscreen creams, age, overweight or obesity [10, 12, 13], or consumption of food containing low quantities of VitD and problems with its absorption [14, 15]. VitD was initially considered a substance capable of regulating calcium and phosphate metabolism [16, 17]. However, recent studies suggest that its effect may be far more multidirectional than previously suspected, and a proper concentration may be necessary to keep organism homeostasis [18–20]. This view was confirmed over time with new reports that showed that most body tissues contain VDR and the enzyme 1-alpha hydroxylase (CYP27B1), which converts VitD into a biologically active form outside kidney tissues [21, 22]. VitD strongly affects the muscular system by stimulating proliferation and cell differentiation, and a low level can cause a reduction in muscle endurance and may be associated with occurrence of myopathy [23, 24]. It has been suggested that some kind of connection may exist between major adverse cardiac events (MACE) and VitD deficiency due to the fact that receptors for vitamin D (VDR) and calcitriol-depedent calcium binding proteins are present on myocardial cells [25–30]. VitD may also be involved in cardiovascular diseases, including atherosclerosis, hypertension, or cardiac hypertrophy. Potential hypotheses on the effects of VitD on cardiovascular disease (CVD) include the downregulation of renin-angiotensin-aldosterone and a direct effect on the heart and vasculature [31, 32]. Over 470 known single nucleotide polymorphisms (SNPs) exist within the *VDR* gene, and this variation may be associated with the incidence and progression of CAD [33–35]. Most research has focussed on the polymorphisms FokI (rs2228570), ApaI (rs7975232), Bsml (rs1544410), and TaqI (rs731236) [35].

In this study, we aimed to determine whether the polymorphisms rs731236, rs7975232, and rs2228570 in the *VDR* gene may be associated with a possible increased risk of CAD incidents in healthy Polish people with a history of P-CAD in their family (FH of P-CAD).

## 2. Materials and Methods

The genetic results presented here were financed by the Medical University of Silesia in Katowice (KNW-2-045/D/8/N), and the patient's anthropometric, biochemical, and lifestyle-related results were collected as part of the MAGNETIC (Metabolic and Genetic Profiling of Young Adults with and without a Family History of Premature Coronary Heart Disease) Study [36].

This study was conducted in accordance with the Declaration of Helsinki and good clinical practice guidelines. The Ethics Committee at the Institute of Occupational Medicine and Environmental Health, Sosnowiec (Resolution no. 03/2013), approved this study and all participants provided informed consent before participation.

### 2.1. Study Population

The study cohort consisted of 845 subjects between 18 and 35 years of age, 386 subjects (45.68%) with a documented angiographically P-CAD incident in their first-degree relatives (relatives <55 years in men and <65 years in women) and 459 (54.32%) subjects in the control group without any documented P-CAD incident in their family. Participants of the study group were offspring of hospitalized P-CAD patients in Silesian Centre for Heart Disease who agreed to telephone contact after the end of their treatment. Samples of case and control groups were recruited both in the spring-summer and autumn-winter periods between July 2015 and October 2017. Signed consent to participate in the study and confirmed medical family history were also required as criteria for inclusion in the study. Exclusion criteria for both groups are acute or chronic disease, pharmacotherapy, pregnancy, lactation, unsure medical family history, age less than 18 and above 35, and lack of informed consent to participate in the study.

### 2.2. IMT Measurement

Intima-media thickness (IMT) measurements were performed as a part of the MAGNETIC study during carotid ultrasonography (USG) with the VIVID E9 system (GE Healthcare) and a 7-14 MHz line probe automatically by the dedicated software during a diastole (R peak) [36].

### 2.3. Biochemical Analyses

Peripheral blood was collected from each patient during the first morning recruitment visit (8-10 hours after the last meal) in S-Monovette tubes with clotting activator (Sarstedt, Germany) and centrifuged at 1500 rpm for 10 minutes at 4°C to obtain serum. Biochemical and immunochemical measurements were made using a Cobas 6000 analyzer (Roche Diagnostics, USA). A full list of reagents used for measurements of metabolites is presented in the Supplementary Materials (Table S1).

### 2.4. DNA Analyses

Whole peripheral blood was collected in S-Monovette tubes with EDTA (Sarstedt, Germany), and DNA was isolated using the GeneMATRIX Quick Blood DNA Purification Kit (Eurx, Poland) and quantitated using a NanoPhotometer™ Pearl spectrophotometer (Implen GmbH, Germany). 4.6 *μ*l of diluted DNA (5 ng/*μ*l) was mixed with 5 *μ*l TaqMan Genotyping Master Mix (Thermo Fisher Scientific, USA) and 0.5 *μ*l 20X TaqMan probe (for either rs731236, rs7975232, or rs2228570) (Thermo Fisher Scientific, USA). For each DNA sample, genotyping for all SNPs was performed separately. In addition, a negative control (NTC) with molecular water was run in the analysis. Genotyping was performed according to the TaqMan probe manufacturer's instructions (Thermo Fisher Scientific, USA) using Cobas z480 (Roche, Switzerland). Using the endpoint genotyping method (analysis of final amplification products), the genotype of each subject was determined from the three *VDR* polymorphisms investigated. ApaI genotyping of three samples failed, and those samples were excluded from further analysis. To further verify the genotyping results, 10% of randomly selected samples were regenotyped. Additionally, three randomly selected samples of all genotypes were analyzed using the Sanger sequencing: amplified fragments containing the polymorphisms were purified using the BigDye XTerminator Purification Kit (Thermo Fisher, USA), sequenced using the BigDye Terminator v3.1 sequencing Kit (Thermo Fisher, USA), and analyzed in the ABI PRISM 3130 Genetic Analyzer (Thermo Fisher, USA) using the Applied Biosystems sequencing analysis software (Thermo Fisher, USA). The genotypes were confirmed in all samples.

### 2.5. Statistical Methods

Statistical analysis was performed using the Power Analysis Software STATISTICA v.13.3 (StatSoft, Krakow, Poland). Clinical data are presented as a mean value ± standard deviation, and nominal values as a percentage of a total count. Comparison of clinical data between control and study groups was performed with *t*-tests and Pearson's chi-square tests. Compatibility of genotype distribution with the Hardy Weinberg equilibrium was performed with the chi-square test and the frequency distribution of *VDR* gene genotypes with the Pearson chi-square compatibility test. Logistic regression analysis was performed to evaluate the association between FH of P-CAD and dominant, codominant, and recessive models of VDR polymorphisms. Analysis of the influence of haplotype on FH of P-CAD was performed using the Fisher exact test. For all tests, a significance level of *p* < 0.05 was designated, and for odds ratios, 95% confidence intervals were calculated.

## 3. Results

Table 1 contains anthropometric, biochemical, and lifestyle-related characteristics of the study cohort separated into the case group (patients with FH of P-CAD) and the control group (patients without FH of P-CAD). The case group consisted of 231 men (59.84%) and 155 women (40.16%) with an average age of 29 years, and the control group of 202 men (44.01%) and 257 women (55.99%) with an average age of 27 years. Patients with P-CAD predisposition had a higher body mass index (BMI) (*p* < 0.001), waist circumference (WC) (*p* < 0.001), and waist-to-hip ratio (WHR) (*p* < 0.001) compared to the control group. Patients with FH of P-CAD had significantly higher levels of fasting glucose (FG) (*p* < 0.001), hemoglobin A1c (HbA1c) (*p* < 0.001), total cholesterol (TC) (*p* = 0.001), low-density cholesterol (LDL) (*p* < 0.001), and triglycerides (TG) (*p* < 0.001), and significantly lower concentrations of high-density cholesterol (HDL) (*p* < 0.001) in comparison to the control group. The mean calcium levels were higher in patients with FH of P-CAD (*p* = 0.001), and mean phosphorus levels were lower (*p* < 0.001). No statistically significant difference was found between the study and control groups in terms of SBP (*p* = 0.449) and DBP (*p* = 0.835). A significant difference in VitD concentration was observed between the study and control groups (*p* = 0.019). A higher concentration of total VitD (25-hydroxyvitamin D (25(OH)D)) was observed in the group of healthy individuals with a family burden towards P-CAD in comparison to the control group (23.50 ± 11.08 vs. 21.76 ± 10.37; *p* = 0.019) (Table 1).

Table 2 demonstrates the genotype distribution of TaqI, ApaI, and FokI polymorphisms in the *VDR* gene in the study and control groups. All the polymorphisms studied were in WHE (*p* > 0.05). For TaqI and ApaI polymorphisms, no significant difference was found between the groups in terms of genotype frequencies (*p* > 0.05). Evaluation of genotype distributions by the chi-square test revealed that only the FokI polymorphism differed significantly between people with or without a family history of P-CAD (*p* = 0.024). As shown in Table 2, the FokI GG genotype was less frequent in individuals with FH of P-CAD than in the control group (27.20% vs. 30.28%). In addition, the AA genotype of FokI polymorphism was significantly more common in the study group compared to the control group (24.61% vs. 16.99%) (Table 2).

The results presented in Table 3 show a potential impact of FokI polymorphism in the *VDR* gene on FH of P-CAD. We considered three models: codominant, dominant, and recessive. Logistic regression analysis showed that in the recessive model, the AA genotype may predispose to potential development of CAD among healthy people over the years (OR: 1.26 (1.07-1.49, *p* = 0.007)) (Table 3). Logistic regression analysis results for TaqI and ApaI polymorphisms are presented as supplementary data (Tables S2 and S3).

The distribution of *VDR* haplotypes shows that the most frequently occurring haplotype in the group with a FH of P-CAD was ACG (24.09%), while in the control group, it was ACA (20.26%). However, no statistically significant difference between those two groups was detected (Table 4).

## 4. Discussion

Cardiovascular disease (CVD) causes 3.9 million deaths in Europe and over 1.8 million deaths in the European Union (EU) each year. The observed increased incidences of CAD in middle-income societies in recent years has caused interest in risk factors associated with this disease [37].

VitD has metabolic activity through the VDR receptor whose gene is located on chromosome 12q12-q14 [38]. VDR binds the active form of VitD (calcitriol), which causes interaction with the retinoid X receptor (*RXR*), thus forming a *VDR*/*RXR*/cofactor complex that binds to the elements of the VitD response element (*VDRE*) in the gene's promoter region. This leads to activation or suppression of expression by interacting with additional coregulators [38–40]. The VDR protein is expressed in many types of cells in the human organism [22]. The *VDR* gene has numerous polymorphisms, including one affecting the site of transcription initiation and thus resulting in formation of alternative isoforms [33, 41]. Confirmation that VDR is present in most cells of the human body suggests that polymorphic changes within it may affect development of many diseases, including CAD [22, 42]. Published studies show a beneficial role of VitD in CVD by regulating factors associated with increased risk for atherosclerosis, such as plasminogen-1 activator inhibitor in human aortic smooth muscle cells [12, 38, 39, 43].

We focused on three single nucleotide polymorphisms of the *VDR* gene, TaqI, ApaI, and FokI. TaqI polymorphism (change from A to G in exon 9) is one of the four most-analyzed polymorphisms of this gene. This polymorphism is localized near an exon-intron border and therefore may potentially affect the stability of *VDR* mRNA [44]. In our analysis, no significant difference was observed in the frequency of TaqI genotypes between the study group of people with FH of P-CAD and the control group (*p* = 0.896). This lack of a significant observational influence of this polymorphism on cardiovascular disease corresponds to other research on this subject; in a Ukrainian study of 118 patients with acute coronary syndrome (ACS), there was also no relationship between ACS and the TaqI polymorphism [45], while He et al. showed no significant differences in the frequency of TaqI polymorphism genotypes between patients with coronary heart disease (CHD) and a control group [34]. Also, in studies involving 137 male patients aged 35-50 with clinically confirmed CAD, there was no relationship between the TaqI genotypes and CAD [46]. In contrast to all other cited studies, logistic regression analysis conducted by Ferrarezi et al. showed association of the allele C TaqI polymorphism with CAD at baseline in a dominant model [47]. Even meta-analyses do not give an unequivocal answer whether TaqI polymorphism can affect CAD development; an analysis by Lu et al. showed that TaqI polymorphism may increase risk of CAD in the analysis of the allelic, dominant, heterozygous, or homozygous models [48], while Alizadeh et al. showed no relationship [41]. Our results indicate that TaqI polymorphism is not related to the development of P-CAD among healthy individuals with a family burden towards this disease.

The second *VDR* polymorphism studied here, the ApaI variant (change from C to A in intron 8) can affect gene expression by altering mRNA stability, altering intronic regulatory elements, or disrupting splicing [44, 49]. We did not observe significant differences in the frequency of ApaI genotypes between the study and the control group (*p* = 0.348), corresponding to the findings of other researchers who reported no association between ApaI and ACS, CHD, or CAD [34, 45, 46]. Also, there are some studies suggesting that *VDR* polymorphisms including ApaI may be associated with the development of diseases such as diabetes or metabolic syndrome [50–53]. Contrary to our results and those of other research teams, Ferrarezi et al. showed that the A allele of the ApaI polymorphism was associated with more frequent CAD in T2DM patients in France [47]; however, in that study, patients with confirmed T2DM were collected, while in our study, healthy people with chronic diseases excluded were recruited. Additionally, meta-analyses of the relationship between ApaI polymorphism and CAD development are contradictory; Lu et al. found a potential protective effect of the A/C genotype of ApaI polymorphism in CAD with T2DM [48], while another meta-analysis showed no such effect, although recognising that in some studies such an association could be seen [41].

Despite similar results regarding the lack of association between TaqI and ApaI polymorphisms and the risk of CAD, it should be noted that only a few published studies on this relationship are available. Of these, almost all are based on a small group of respondents from different populations (Caucasian, African, and Asian), which is undoubtedly a problem in interpreting the results. This polymorphism requires additional research to determine if it may be related to CAD.

The third *VDR* gene polymorphism analyzed here was FokI (rs10735810, merged into rs2228570, a change from A to G in exon 2). This polymorphism in the translated region is the only one in this study which causes functional and structural variability of the VDR protein and may cause a longer or shorter VDR protein variant [54]. FokI polymorphism is characterized by the presence of two ATG start codons separated by six nucleotides. If the A allele (f allele) is present, a long f-VDR protein (427 aa) with lower activity is formed. In the case of substitution by the G allele (F allele), we observe only one start codon which results in the formation of a shorter F-VDR protein (424 aa) [55–57]. Depending on the resulting form of the VDR protein (long or short), different ability to induce transcription of VitD-dependent genes is observed [42]. Additionally, carriers of the GG gene variant may have greater VDR activity than carriers of the GA or AA gene variant [56].

In our study, only the distribution of FokI genotypes of the *VDR* gene was significantly different between healthy people with FH of P-CAD and those without (*p* = 0.024). In addition, we show that the AA genotype of FokI polymorphism was significantly more common in the group of healthy people with a family burden towards P-CAD compared to the control group (24.61% vs. 16.99%), and the FokI GG genotype was less common in people with P-CAD family burden compared to people in the control group (27.20% vs 30.28%). According to the literature, we can assume that carriers of the AA variant *VDR* gene may have less VDR activity than carriers of the GA or GG gene variant, which may affect the change in expression of VitD-dependent genes and contribute to the potential inflammation and development of CAD in the future [34, 35, 56]. Moreover, meta-analysis shows an interesting relationship between FokI polymorphism and CAD and indicates that the C (G) allele and the GG genotype could have a protective effect in CAD [48]. GG genotype of FokI polymorphism showed a significant difference between CHD patients and controls in study conducted by He et al. in Chinese population (28.4% vs. 41.8%, *p* = 0.039). GG genotype frequency of CHD patients was significantly lower. The AA genotype of FokI polymorphism was more common in the CHD group compared to the control group (23.7% vs. 17.9%, *p* = 0.319) [34]. Jun et al. also showed that risk of CHD was higher in carriers with the T (A) allele of FokI polymorphism (*p* < 0.001) [54]. Our results indicate that the AA genotype may affect the risk of CAD in a recessive model (OR: 1.26 (1.07-1.49, *p* = 0.007)). Additionally, analysis of 760 patients classified for CAD shows that FokI polymorphism may be a functional *VDR* polymorphism in the process of collateralization, whose range was highest in the FF (GG) genotype and the lowest in the ff (AA) genotype [42]; however, there is no scientific consensus concerning the role of this polymorphism in CAD. Some studies suggest that there is no association between FokI and ACS in a Ukrainian population and CAD in a Chinese population [45, 58]. It should be noted that most publications relating to the association of FokI genotypes with selected CVDs are consistent with the results of our study cohort.

In addition, we present polymorphisms in the form of haplotype distribution (TaqI-ApaI-FokI) within the study and control groups, which, according to the data, can improve statistical performance and interpretation of genetic studies on the relationships between polymorphisms and risk of developing the disease [59]. The ACG haplotype was more common in our study population (24.09%) than in the control population (19.61%). The most common haplotype in the control group was the ACA haplotype (20.24%) than in the study group (18.39%). VDR haplotype frequencies did not differ significantly between the control and study populations. To our knowledge, there are currently no published haplotype analyses for TaqI, ApaI, and FokI polymorphisms regarding FH of P-CAD.

We decided to introduce validity of analysis of *VDR* polymorphisms haplotypes in other diseases. Zhong et al. in study of Chinese patients with type 2 diabetes mellitus (T2DM) showed that the TAA haplotype (FokI-BsmI-ApaI) was significantly more common in diabetic retinopathy (DR) patients than in those without DR (OR: 1.65, 95% CI: 1.01-2.70; *p* = 0.04) [60]. In addition, the AAC haplotype (BsmI-ApaI-TaqI) was associated with an increased risk of CAD in T2DM patients compared with the GCT haplotype (OR: 1.12, 95% CI: 1.02–1.28; *p* = 0.04) [47]. Haplotype analysis of *VDR* gene polymorphisms was also carried out in studies of type 1 diabetes mellitus (T1DM). Rasoul et al. showed that the fCTb and fCTB haplotypes of FokI-TaqI-ApaI-BsmI polymorphisms were more common in the study group. These haplotypes may be associated with T1DM in Kuwaiti children (OR: 4.81, 95% CI: 1.63-14.24; *p* = 0.002 and OR: 7.07, 95% CI: 2.93-17.07; *p* < 0001) [61]. In contrast, lack of association between FokI-BsmI-ApaI-TaqI polymorphisms haplotypes with T1DM in a Portuguese population was demonstrated by Lemos et al. [62]. Haplotype analysis of *VDR* gene polymorphisms (FokI-BsmI-ApaI-TaqI) has also been carried out in metabolic syndrome (MetS), where it was demonstrated that the CATT haplotype was associated with an increased risk of MetS (OR: 4.32, 95% CI: 1.32-14.10; *p* = 0.016), while the TGGT haplotype reduced the risk of MetS occurring in a Thai population (OR: 0.68, 95% CI: 0.48-0.98; *p* = 0.042) [53]. It should be noted that results of haplotype analyses most often concern small population numbers, which may limit (affect) the significance (incorrect interpretation) of the conclusions. It would be necessary to study a larger group to see if the haplotypes of selected *VDR* gene polymorphisms could have an impact on the potential increased risk of premature CAD.

In our opinion, the results of our tests should be reexamined (verified) after several decades to determine if CAD has developed among people in the study group. It should also be noted that the available publications concerning *VDR* polymorphisms and CAD or P-CAD are relatively limited both in number and in size of the group studied, CVDs, polymorphism selected, and age and population, which undoubtedly impacts their interpretation and comparability.

## 5. Conclusions

This study documents a potential connection between FokI polymorphism of the *VDR* gene and susceptibility to P-CAD in a healthy population with family history of this disease. TaqI and ApaI polymorphisms show no significant relationship with FH of P-CAD. Our research on FokI, TaqI, and ApaI polymorphisms should be verified as a part of a prospective assessment to check whether the study group was actually exposed to more frequent CAD occurrences.

## Figures and Tables

**Table 1:** Characteristics of study and control populations.

Parameters	Study population with FH of P-CAD *n* (%) 386 (45.68)	Control group *n* (%) 459 (54.32)	*p* values
Physical activity			0.932
Small	97 (25.13)	119 (25.93)	
Moderate	184 (47.67)	213 (46.41)	
High	105 (27.20)	127 (27.67)	
DM2 family history	84 (21.76)	42 (9.15)	<0.001
Cigarette smoking, *n* (%)			0.002
Smoking	103 (26.68)	83 (18.24)	
No smoking	237 (61.40)	334 (73.41)	
Smoking in the past	46 (11.92)	37 (8.13)	

	Mean ± SD		

WC (cm)	85.3 ± 13.5	78.8 ± 13.2	<0.001
BMI (kg/m^2^)	25.3 ± 4.6	23.4 ± 4.1	<0.001
WHR	0.9 ± 0.1	0.8 ± 0.1	<0.001
SBP (mmHg)	126.6 ± 13.6	125.9 ± 14.4	0.449
DBP (mmHg)	78.5 ± 10.8	78.3 ± 10.1	0.835
Carotid intima media			
Thickness (mm)			
Right (average)	0.53 ± 0.08	0.52 ± 0.06	0.010
Left (average)	0.54 ± 0.09	0.51 ± 0.08	<0.001
Cholesterol (mmol/l)	5.08 ± 1.08	4.85 ± 0.99	0.001
LDL (mmol/l)	3.16 ± 0.97	2.76 ± 0.88	<0.001
HDL (mmol/l)	1.54 ± 0.44	1.68 ± 0.49	<0.001
Triglycerides (mmol/l)	1.31 ± 1.33	1.06 ± 0.65	<0.001
Fasting glucose (mmol/l)	5.10 ± 0.47	4.82 ± 0.40	<0.001
HbA1C (%)	5.06 ± 0.27	4.88 ± 0.24	<0.001
Vitamin D (ng/ml)	23.50 ± 11.08	21.76 ± 10.37	0.019
Calcium (mmol/l)	2.43 ± 0.09	2.41 ± 0.08	0.001
Phosphorous (mmol/l)	1.05 ± 0.16	1.14 ± 0.16	<0.001

**Table 2:** Genotypes frequencies of TaqI, ApaI, and FokI polymorphisms.

Genotypes	All *n* (%)	Study population with FH of P-CAD *n* (%)	Control group *n* (%)	*p* value^a^
TaqI (rs731236)	845 (100)	386 (45.68)	459 (54.32)	
AA	340 (40.24)	152 (39.38)	188 (40.96)	0.896
AG	389 (46.04)	180 (46.63)	209 (45.53)	
GG	116 (13.73)	54 (13.99)	62 (13.51)	
*p* value^b^	0.778	0.951	0.746	

ApaI (rs7975232)	842 (100)	386 (45.84)	456 (54.16)	
CC	222 (26.37)	93 (24.09)	129 (28.29)	0.348
AC	409 (48.57)	196 (50.78)	213 (46.71)	
AA	211 (25.06)	97 (25.13)	114 (25.00)	
*p* value^b^	0.411	0.758	0.167	

FokI (rs2228570)	845 (100)	386 (45.68)	459 (54.32)	
AA	173 (20.47)	95 (24.61)	78 (16.99)	0.024
AG	428 (50.65)	186 (48.19)	242 (52.72)	
GG	244 (28.88)	105 (27.20)	139 (30.28)	
*p* value^b^	0.557	0.484	0.116	

^a^ *p* value of Pearson's chi-square test. ^b^*p* value of chi-squared test for HWE.

**Table 3:** FokI polymorphism of the *VDR* gene and FH of P-CAD.

FokI (rs2228570)	All *n* (%) 845 (100)	Study population with FH of P-CAD *n* (%) 386 (45.68)	Control group *n* (%) 459 (54.32)	OR (95% CI)	*p* value
Codominant					
AA	173 (20.47)	95 (24.61)	78 (16.99)	1.27 (1.04-1.55)	0.017
AG	428 (50.65)	186 (48.19)	242 (52.72)	1.01 (0.86-1.18)	0.915
GG	244 (28.88)	105 (27.20)	139 (30.28)	1.00 (ref.)	

Dominant					
AG + AA	601 (71.12)	281 (72.80)	320 (69.72)	1.08 (0.93-1.25)	0.325
GG	244 (28.88)	105 (27.20)	139 (30.28)	1.00 (ref.)	

Recessive					
AA	173 (20.47)	95 (24.61)	78 (16.99)	1.26 (1.07-1.49)	0.007
AG + GG	672 (79.53)	291 (75.39)	381 (83.01)	1.00 (ref.)	

**Table 4:** *VDR* haplotype frequencies in healthy individuals with FH of P-CAD and controls.

Haplotypes TaqI-ApaI-FokI	Study population with FH of P-CAD *n* (%) 386 (45.68)	Control group *n* (%) 459 (54.32)	*p* value	OR
AAA	34 (8.81)	50 (10.89)	0.356	1.27 (0.80-2.00)
AAG	48 (12.44)	55 (11.98)	0.916	0.96 (0.63-1.45)
ACA	71 (18.39)	93 (20.26)	0.541	1.13 (0.80-1.59)
ACG	93 (24.09)	90 (19.61)	0.131	0.76 (0.55-1.05)
GAA	47 (12.18)	60 (13.07)	0.756	1.06 (0.71-1.60)
GAG	59 (15.28)	67 (14.60)	0.846	0.95 (0.65-1.38)
GCA	14 (3.63)	21 (4.58)	0.604	1.19 (0.60-2.33)
GCG	20 (5.18)	23 (5.01)	1.000	0.97 (0.52-1.79)

## Data Availability

The data used to support the findings of this research are available from the corresponding author upon request.

## Supplementary Materials

Supplementary Materials Table S1: List of reagents. Table S2: TaqI polymorphism of the *VDR* gene and FH of P-CAD. Table S3: ApaI polymorphism of the *VDR* gene and FH of P-CAD.
