# Supplementary material for: VDR Gene Polymorphisms in Healthy Individuals with Family History of Premature Coronary Artery Disease
Source: Dis Markers. 2021 Jan 29;2021:8832478. doi: 10.1155/2021/8832478 (PMC7867440; doi:10.1155/2021/8832478)
Supplement: Supplementary Materials — Table S1: List of reagents. Table S2: TaqI polymorphism of the VDR gene and FH of P-CAD. Table S3: ApaI polymorphism of the VDR gene and FH of P-CAD. [file 8832478.f1.doc]

Table S1: List of reagents.

| **Laboratory tests** | **Test name, company** | **Catalog number** |
| --- | --- | --- |
| Total cholesterol | CHOL2, Roche Diagnostics, Indianapolis, IN, USA | 03039773190 |
| Glucose | GLUC3, Roche Diagnostics, Indianapolis, IN, USA | 04404483190 |
| HbA1C | A1C-3 (TQ), Roche Diagnostics, Indianapolis, IN, USA | 05336163190 |
| High density lipoprotein | HDLC3, Roche Diagnostics, Indianapolis, IN, USA | 04399803190 |
| Low density lipoprotein | LDL_C, Roche Diagnostics, Indianapolis, IN, USA | 03038866322 |
| Triglycerides | TRIGL, Roche Diagnostics, Indianapolis, IN, USA | 20767107322 |
| Vitamin D | Vitamin D Total Cs Elecsys, Roche Diagnostics, Indianapolis, IN, USA | 05894913190 |
| Calcium | CA 2, Roche Diagnostics, Indianapolis, IN, USA | 05061482190 |
| Phosphorous | PHOS 2, Roche Diagnostics, Indianapolis, IN, USA | 03183793122 |
|  |  |  |
|  |  |  |
|  |  |  |
|  |  |  |

Table S2: TaqI polymorphism of the *VDR* gene and FH of P-CAD.

| **TaqI (rs731236)** | **All**  **n (%)**  **845 (100)** | **Study population with FH of P-CAD**  **n= (%)**  **386 (45.68)** | **Control group**  **n= (%)**  **459 (54.32)** | **OR (95%Cl)** | **p-value** |
| --- | --- | --- | --- | --- | --- |
| Co-dominant |  |  |  |  |  |
| AA | 340 (40.24) | 152 (39.38) | 188 (40.96) | 0.96 (0.78-1.19) | 0.730 |
| AG | 389 (46.04) | 180 (46.63) | 209 (45.53) | 0.99 (0.81-1.22) | 0.958 |
| GG | 116 (13.73) | 54 (13.99) | 62 (13.51) | 1.00 (ref.) |  |
| Dominant |  |  |  |  |  |
| AG + GG | 505(59.76) | 234 (60.62) | 271 (59.04) | 1.03 (0.90-1.86) | 0.641 |
| AA | 340 (40.24) | 152 (39.38) | 188 (40.96) | 1.00 (ref.) |  |
| Recessive |  |  |  |  |  |
| GG | 116 (13.73) | 54 (13.99) | 62 (13.51) | 1.02 (0.84-1.24) | 0.839 |
| AG + AA | 729 (86.27) | 332 (86.01) | 397 (86.49) | 1.00 (ref.) |  |

Table S3: ApaI polymorphism of the *VDR* gene and FH of P-CAD.

| **ApaI (rs7975232)** | **All**  **n (%)**  **842 (100)** | **Study population with FH of P-CAD**  **n (%)**  **386 (45.84)** | **Control group**  **n (%)**  **456 (54.16)** | **OR (95%Cl)** | **p-value** |
| --- | --- | --- | --- | --- | --- |
| Co-dominant |  |  |  |  |  |
| AA | 211 (25.06) | 97 (25.13) | 114 (25.00) | 1.00 (ref.) |  |
| AC | 409 (48.57) | 196 (50.78) | 213 (46.71) | 1.04 (0.88-1.23) | 0.645 |
| CC | 222 (26.37) | 93 (24.09) | 129 (28.29) | 0.92 (0.76-1.11) | 0.393 |
| Dominant |  |  |  |  |  |
| AC + CC | 631 (74.94) | 289 (74.87) | 342 (75.00) | 0.99 (0.85-1.17) | 0.966 |
| AA | 211 (25.06) | 97 (25.13) | 114 (25.00) | 1.00 (ref.) |  |
| Recessive |  |  |  |  |  |
| CC | 222 (26.37) | 93 (24.09) | 129 (28.29) | 0.90 (0.77-1.05) | 0.169 |
| AC + AA | 620 (73.63) | 293 (75.91) | 327 (71.71) | 1.00 (ref.) |  |
